# Mental health symptom burden in elite ice hockey players and its association with self-reported concussive events

**DOI:** 10.1186/s13102-024-00989-0

**Published:** 2024-09-23

**Authors:** Mitchell J. Andersson, Göran Kenttä, Emma Claesdotter-Knutsson, Anders Håkansson

**Affiliations:** 1 Department of Clinical Sciences Lund, Psychiatry, Lund University, Lund, Sweden; 2 Clinical Sports and Mental Health Unit, Malmö Addiction Center, Region Skåne, Malmö, Sweden; 3 The Swedish School of Sport and Health Sciences, Stockholm, Sweden; 4 The Swedish Sports Confederation, Stockholm, Sweden; 5 School of Human Kinetics, University of Ottawa, Ottawa, ON Canada; 6 Child and Adolescent Psychiatry Outpatient Clinic, Region Skåne, Lund, Sweden

**Keywords:** Sports-related concussion, Elite athletes, Alcohol use, Social media use, Depression, Anxiety, Burnout

## Abstract

**Background:**

Some studies suggest that elite athletes experience adverse mental health symptoms at rates commensurate with the general population, despite the well-established buffering effects of exercise. Within contact sports, such as ice-hockey, recurrent concussions may be a source of this discrepancy. We compared the point prevalence of various mental health outcomes with other athlete and general population samples, as well as investigated their relationship with concussive events.

**Methods:**

We surveyed 648 active ice hockey players from the top two men’s tiers and the top women’s tier in Swedish elite ice hockey on lifetime concussive events, hazardous alcohol use, problematic social media use, depression, anxiety, and burnout.

**Results:**

Hazardous alcohol use was more prevalent among male ice hockey players (29.5% AUDIT-C ≥ 6) compared to other athlete and general population samples, while other mental health symptoms were less common. Female ice hockey players reported higher hazardous alcohol consumption (36.4% AUDIT-C ≥ 4) than another athlete sample and more burnout (19.1%) than the general population. After adjusting for covariates, athletes with 3+ concussive events had 2.1 times the odds of elevated depressive symptoms and 3.5 times the odds of elevated burnout symptoms compared to those with no concussion history. Treating lifetime concussive events as a continuous predictor revealed positive correlations with all outcomes except for hazardous alcohol use.

**Conclusions:**

Mental health outcome rates among active elite ice hockey athletes differ from those of other athlete and general population samples, whilst concussive events may be particularly linked to elevated symptoms of depression and burnout.

**Supplementary Information:**

The online version contains supplementary material available at 10.1186/s13102-024-00989-0.

## Introduction

Despite the demonstrated protective effects of sports participation and exercise against adverse mental health symptoms like depression [1, 2], some studies indicate that elite athletes are just as susceptible to developing various mental health issues as the general population [3]. This disparity has prompted extensive interdisciplinary research to uncover the underlying causes of their mental health challenges. Elite athletes encounter unique stressors and barriers that often preclude them from acknowledging or seeking treatment for their symptoms [4, 5]. This issue may be particularly pronounced in ice hockey, where stigma surrounding mental health problems has historically contributed to their concealment and exacerbation [6, 7]. Alongside psychosocial factors, ice hockey is a contact sport that exposes athletes to the risk of serious injuries, such as recurrent concussions, which may contribute to the development of psychopathology [8, 9].

Though rates of mental health symptoms among active elite athletes may mirror that of the general population for certain outcomes [3, 10–12], there is a growing call for research assessing the impact of multiple concussions on the long-term mental health of elite athletes [13]. Concussion falls on the spectrum of traumatic brain injury and occurs as a result of an impulsive force that is transmitted to the head and frequently initiates a biochemical cascade in the brain that causes temporary neurological dysfunction [14–16]. Most athletes who sustain a sports-related concussion recover completely within one month [16], yet some research indicates that neuropsychological symptoms may persist beyond the acute phase of injury [13, 17].

Research on the cumulative effects of recurrent concussions has often focused on retired athletes, with some studies indicating that a greater number of previous concussions (concussion history; CHx) correlates with symptoms of depression and anxiety [18–21]. However, it is unclear whether these symptoms develop after retirement or are present during the athlete’s playing career [18]. Few large-scale studies have examined the relationship between CHx and mental health in active elite collision sport athletes, leaving this question inadequately addressed in the literature. Additionally, it is uncertain whether other mental health outcomes, such as addictive disorders or burnout, are similarly elevated.

Depression and anxiety are among the most frequently studied symptoms in the concussion literature [19], believed to arise from concussion-induced neurodegeneration, neural network disruption, and injury-related ecological factors [9, 15]. Findings from studies conducted among active elite athletes measuring their relationship with CHx are inconsistent. A repeated cross-sectional survey by du Preez and colleagues [22] found that among 404 active elite male rugby league players, those with three or more concussions had twice the odds of moderate-to-severe depression at pre-season compared to those with two or fewer concussions, controlling for psychiatric history, age, ethnicity, and injury status. Despite a larger adjusted odds ratio for the in-season sample, this relationship did not remain significant, likely due to inadequate power. In another cross-sectional study of 508 active female elite athletes, Jónsdóttir and colleagues [23] found those who reported two or more concussions had between 3.5- and 4.9-times higher odds of screening for moderate-to-severe symptoms of depression and between 3.4- and 3.5-times higher odds of screening for moderate-to-severe symptoms of anxiety. These enlarged effect sizes are not surprising as female athletes tend to report greater post-concussion symptom burden compared to male athletes [24, 25]. Lastly, a cross-sectional survey administered to 749 male and female elite athletes in Australia indicated that only those who had sustained two concussions during their lifetime endorsed elevated mental health symptoms and psychological distress, not those who reported one nor three or more concussions [26]. Because only 2.5% of their sample had incurred three or more concussions during their careers, they likely did not have ample statistical power to report a meaningful odds ratio.

In contrast to the breadth of studies on depression and anxiety, no studies hitherto have investigated CHx’s association with burnout in elite athlete samples. Burnout is characterized by an athlete’s inability to cope with stressors within their environment and its etiology is multidimensional and complex [27]. Previous research suggests that concussive events may disrupt the psychophysiological stress response [28], which may serve as a precursor to the development of burnout in athletes. Similar to the dearth of research on burnout, few studies have examined the association between CHx and addictive behaviors among active elite athletes. These behaviors may arise from similar biomechanical mechanisms as depression and anxiety [29] or through self-medication to cope with post-concussion symptoms [30]. A prospective cohort study among 573 elite rugby players by Kilic and colleagues [31] found that the number of concussions sustained over the past year was associated with a 1.5-fold increase in the odds of endorsing alcohol misuse while controlling for age and family history of psychiatric disorder. Questions have also arisen about social media addiction among athletes, with some elites struggling to balance their social media use during major sporting events [32]. To our knowledge, no published studies have investigated problematic social media use quantitatively in elite athletes, nor its connection with CHx. Addictive behaviors can have a myriad of negative consequences during an athlete’s sporting career [33–35] and can perpetuate poor mental health into retirement from sport [36].

In a cohort of both male and female Swedish elite ice hockey players, we aimed to describe and compare their mental health symptom burden with other athlete and general population samples, and to assess the association between symptom burden and self-reported concussive events. We hypothesized based on extant research that (1) our sample would have similar rates of mental health symptoms compared to other athlete and general population samples and (2) participants with more previous self-reported concussive events would have higher odds of endorsing elevated symptoms compared to those with fewer or with no previous history of concussive events.

## Materials and methods

This cross-sectional study surveyed current elite ice hockey players competing in the Swedish Hockey League (SHL; Men’s Tier I), HockeyAllsvenskan (HA; Men’s Tier II), and the Swedish Women’s Hockey League (SWHL; Women’s Tier I) using a self-administered online questionnaire. Before submitting any demographic information or responding to study items, all participants provided digital informed consent. The study procedures adhered to the Declaration of Helsinki and was approved by the Swedish Ethical Review Authority (Dnr: 2019–03393).

### Participants

During scheduled informational meetings between the Player’s Union (Sveriges Ishockeyspelares Centralorganisation; SICO) and the players, one of the researchers or a SICO representative approached and presented the study to all athlete attendees. Participants met the following inclusion criteria: (1) being an active team member of a SHL, HA, or SWHL team, (2) being 16 years old or older, (3) and being able to comprehend survey instructions and read survey texts in English or Swedish. We made study visits to 27 of the 28 teams competing in the SHL and HA during the 2022/2023 season between August 31st, 2022 and February 8th, 2023, and visited all 10 teams participating in the SWHL during the 2023/2024 season between January 25th and February 27th, 2024. The majority of visits occurred during the regular season, with the remainder taking place during the pre-season.

### Procedure

Before providing a QR code for players to scan with their mobile phones that directed them to the online survey, we described the study’s objectives and assured players that their responses would be kept anonymous and confidential. A researcher was either present to answer questions about the study and concussion, or respondents were instructed to email the primary investigator via an email address listed on the survey’s landing page. If a researcher or SICO representative was unable to present the study, players were directed to a video in which a researcher described the study and provided background information about the diagnosis of concussion. The questionnaire collected relevant demographic information, lifetime incidence of concussive events, psychiatric history, and screening tools assessing current symptoms of hazardous alcohol use, problematic social media use, depression, anxiety, and burnout. If a participant met the clinical cut-off for any of the screening instruments, they were notified of this result and provided with contact information for healthcare providers in Sweden who specialize in treating elite athletes.

### Instrument

The questionnaire developed for this study was created in both Swedish and English using validated measures of mental health outcomes in collaboration with physicians, psychiatrists, sports medicine professionals, and research psychologists with ice hockey playing experience (see Additional file 1). The survey was pilot tested and improved based on the feedback of seven current semi-elite and one former elite ice hockey players. Participants selected English or Swedish as the survey language before being redirected to a page containing study information and the consent form. The participants then indicated their sex (male/female) and age (25 or younger/26 or older). We collected participant psychiatric history by asking whether they had ever been diagnosed with attention deficit hyperactivity disorder (ADHD), a learning disorder (e.g., dyslexia), depression, panic disorder, social phobia, burnout, or obsessive compulsive disorder.

#### Main exposure: self-reported lifetime concussive events

Concussion is a clinical diagnosis that frequently goes unreported for sundry psychosocial reasons in athlete samples [9], calling into question the validity of measuring only diagnoses from a medical professional. Therefore, we defined CHx in the present study using self-reported lifetime concussive events that resulted in common clinical signs and symptoms of concussion [14] as a proxy. We asked participants on how many occasions they had experienced (1) balance problems or dizziness without experiencing amnesia or falling unconscious, (2) anterograde or retrograde amnesia without falling unconscious, (3) a period of unconsciousness under thirty seconds, or (4) a period of unconsciousness longer than thirty seconds after a blow to the head or neck. The scale provided for each item ranged from 0 to 4 + occasions, from which we calculated each player’s self-reported lifetime concussive impact history by taking the sum reported for each post-collision experience, weighing each occasion equally where the maximum was 16. Based on previous research [37], we analyzed CHx as continuous and as stratified groups: those with no CHx (0), those who had self-reported one or two concussive events (1–2), and those who reported three or more concussive events (3+).

#### Main outcomes

##### Hazardous alcohol use

We employed the Alcohol Use Disorder Identification Test – Consumption (AUDIT-C) to assess levels of hazardous drinking [38]. The AUDIT-C is an alcohol consumption measure consisting of three items that assess drinking frequency, volume, and binge drinking frequency. Responses are given on three unique ordinal scales that are scored from 0 to 4, with cumulative scores ranging from 0 to 12. In accordance with Lundin and colleagues’ [39] validation of the AUDIT-C in Swedish adults, more conservative cut-offs were used for male (≥ 6) and female (≥ 4) athletes. Their study demonstrated that the AUDIT-C performs similarly well to the full AUDIT questionnaire, with excellent sensitivity and specificity in identifying alcohol use disorders and dependence. Internal consistency for the AUDIT-C was adequate in our study (α = 0.69).

#### Problematic social media use

We administered the Bergen Social Media Addiction Scale (BSMAS) to assess levels of problematic social media use [40]. The BSMAS consists of six items measuring “salience, conflict, mood modification, withdrawal, tolerance, and relapse” [40, pp.9] using six-point Likert-type scale ranging from 0 (never) to 5 (very often). Cumulative scores in the present study ranged from 0 to 30. In accordance with Zarate and colleagues [41], the cut-off for at-risk social media use was set to 14 and then clinical cut-off for problematic social media use was set to 26. The BSMAS scale has been demonstrated to be a valid and reliable measure of problematic social media use in Western adult samples [41] and has been previously translated and implemented in a Swedish context [42]. Internal consistency for the BSMAS was good in our study (α = 0.84).

#### Depression

We administered the Patient Health Questionnaire – 9 (PHQ-9) to assess past two-week depression symptom severity [43]. The PHQ-9 consists of nine items measuring somatic (sleep, fatigue, appetite, concentration, psychomotor agitation) and non-somatic (anhedonia, depressed mood, worthlessness, thoughts of death) symptoms based on the *Diagnostic and Statistical Manual of Mental Disorders*,* Fourth Edition* [DSM-IV; 44–45]. Responses are given on a Likert-type scale ranging from 0 (not at all) to 3 (nearly every day), leading to cumulative scores ranging from 0 to 27. Participants scoring between 5 and 9 were considered as having mild depressive symptoms, while those scoring 10 or more were considered as having moderate-to-severe depressive symptoms. It has been validated in a sample of American college athletes [46] and the Swedish-translated version has been shown to be a valid and reliable measure of depression in an adult sample of primary care patients and psychiatric outpatients [47]. Internal consistency for the PHQ-9 was good in our study (α = 0.85).

### Anxiety

We used the Generalized Anxiety Disorder Scale – 7 (GAD-7) to assess past two-week anxiety symptom severity [48]. The GAD-7 consists of seven items measuring nervousness, anxiety, worried thoughts, difficulty relaxing, restlessness, irritability, and catastrophizing based on DSM-IV criteria for generalized anxiety disorder [44]. Participants respond on a scale ranging from 0 (not at all) to 3 (nearly every day), leading to cumulative scores ranging from 0 to 21. Participants scoring between 5 and 9 were considered as having mild symptoms of anxiety, while those scoring 10 or more were considered as having moderate-to-severe symptoms of anxiety. It has been validated in a sample of American student-athletes [49] and the Swedish-translated version has been used in previous studies but has not been officially validated [50]. Internal consistency for the GAD-7 was good in our study (α = 0.87).

### Burnout

We used the Gothenburg Institute of Stress Medicine’s Self-Reported Exhaustion Syndrome (s-ES) questionnaire to measure symptoms of burnout, which is based on the Swedish National Board of Health and Welfare’s diagnostic criteria for stress-related burnout disorder [51]. The s-ES consists of three parts: Part A assesses exhaustion and its relation to long-term stress with two items. Part B includes six items examining burnout symptoms such as concentration difficulties, performance under pressure, irritability, physical exhaustion, somatic symptoms, and sleep disturbance. Part C consists of one item evaluating the impact of these symptoms on well-being and functionality. Items from Parts A and B are answered on a binary scale and Part C is answered on an ordinal scale (“No, not at all; Yes, somewhat; Yes, to a great extent”). A positive screen for burnout requires both items in Part A to be true, four of six symptoms from Part B to be present, and acknowledgement from the participant that these symptoms have affected them to some degree in Part C. For this study, we added a subclinical cutoff to identify at-risk individuals: participants who affirmed one or both items in Part A and reported experiencing at least three symptoms from Part B. The s-ES has been previously utilized in studies among Swedish workers on sick-leave with common mental disorders [52] as well as working adults in the Swedish general population [53]. Both the Swedish and English versions are available online [51].

### Statistical analysis

Numeric variables are summarized using means and standard deviations or medians and interquartile ranges, and categorical variables are summarized using counts and percentages. We conducted Chi-square (χ^2^) and Fisher exact tests to compare rates of mental health outcomes between the present study and other studies employing the same screening measures. To enable comparison, *post hoc* adjustments were made to our chosen cutoffs to match those used in other studies conducted in athlete and general population samples. Bivariate relationships between concussive events and symptom burden on a continuous scale were assessed using analyses of variance (ANOVA) or nonparametric Kruskal-Wallis and Bonferroni-corrected Dunn tests for each respective sex separately. To assess the relationship between self-reported concussive events and endorsing any level of outcome (mild-to-severe), we conducted binary logistic regressions and adjusted for multiple comparisons using Tukey corrections. We generated univariate and multivariate models adjusted for previous LD/ADHD diagnoses, age, and sex. CHx and sex interaction terms were introduced to determine if effects differed by sex. Effects are reported as odds ratios (OR) with 95% confidence intervals (CI). We used R-V4.2.1 to conduct analyses and create Figs. [54–58].

## Results

### Participants

In the present study, 648 participants were included. The preponderance was male (72.2%), completed the survey in Swedish (85.5%), and 25 years old or younger (53.4%). Our male athlete sample represented 55.7% (468/840) of SHL and HA players from the 2022–2023 season, while our female athlete sample represented 77.9% (180/231) of SWHL players from the 2023–2024 season [59]. Sample characteristics, both overall and stratified by sex, are presented in Table 1.

**Table 1:** Sample characteristics by sex

	Sex			
Variable	Male (*n* = 468)	Female (*n* = 180)	Total (*N* = 648)	
**Demographic**				
Age, *n* (%)				
≤ 25	206 (44.0%)	140 (77.8%)	346 (53.4%)	
26+	262 (56.0%)	40 (22.2%)	302 (46.6%)	
Language, *n* (%)				
Swedish	447 (95.5%)	107 (59.4%)	554 (85.5%)	
English	21 (4.5%)	73 (40.6%)	94 (14.5%)	
**Previous Concussive Events**				
Total	1.0 [0.0, 3.0]	0.0 [0.0, 2.0]	1.0 [0.0, 2.0]	
0	212 (45.8%)	89 (50.6%)	301 (47.1%)	
1–2	128 (27.6%)	55 (31.3%)	183 (28.6%)	
3+	123 (26.6%)	32 (18.2%)	155 (24.3%)	
Missing, *n*	5	4	9	
**Current Drug Use**				
Illicit drugs past year, *n* (V%)	13 (2.9%)	16 (9.2%)	29 (4.6%)	
Missing, *n*	16	7	23	
Analgesics past month, *n* (V%)	28 (6.2%)	5 (2.9%)	33 (5.3%)	
Missing, *n*	16	7	23	
Soporifics past month, *n* (V%)	57 (12.6%)	24 (13.9%)	81 (13.0%)	
Missing, *n*	16	7	23	
**Psychiatric Diagnoses**				
ADHD/LD Dx, *n* (V%)	22 (4.8%)	29 (16.7%)	51 (8.1%)	
Missing, *n*	11	6	17	
Psychiatric Dx, *n* (V%)	29 (6.3%)	46 (26.4%)	75 (11.9%)	
Missing, *n*	11	6	17	
GD Dx, *n* (V%)	12 (2.6%)	0 (0.0%)	12 (1.9%)	
Missing, *n*	11	6	17	
SUD Dx, *n* (V%)	7 (2.1%)	6 (3.4%)	13 (1.6%)	
Missing, *n*	11	6	17	
**Psychiatric Outcomes**				
Alcohol: AUDIT-C, *M* ± *SD*	4.5 ± 1.8	2.7 ± 1.9	4.0 ± 2.0	
Hazardous consumption, *n* (V%)	134 (29.5%)	63 (36.4%)	197 (31.4%)	
Missing, *n*	13	7	20	
Social media: BSMAS, *Mdn* [Q1-Q3]	4.0 [1.0, 8.0]	7.0 [3.0, 11.0]	4.0 [1.0, 9.0]	
At-risk 14 ≤ 25, *n* (V%)	37 (8.2%)	26 (15.0%)	63 (10.1%)	
Problematic use ≥ 26, *n* (V%)	0 (0.0%)	2 (1.2%)	2 (0.3%)	
Missing, *n*	19	7	26	
Depression: PHQ-9, *Mdn* [Q1-Q3]	2.0 [0.0, 3.0]	4.0 [2.0, 7.0]	2.0 [0.0, 5.0]	
Mild 5 ≤ 9, *n* (V%)	71 (15.8%)	55 (31.8%)	126 (20.2%)	
Moderate/Severe ≥ 10, *n* (V%)	7 (1.6%)	26 (15.0%)	33 (5.3%)	
Missing, *n*	18	7	25	
Anxiety: GAD-7, *Mdn* [Q1-Q3]	2.0 [0.0, 3.0]	4.0 [2.0, 7.0]	2.0 [0.0, 5.0]	
Mild 5 ≤ 9, *n* (V%)	69 (15.3%)	52 (30.1%)	121 (19.4%)	
Moderate/Severe ≥ 10, *n* (V%)	7 (1.6%)	18 (10.4%)	25 (4.0%)	
Missing, *n*	17	7	24	
Burnout: s-ES, *n* (V%)				
At-risk, see caption for conditions*	29 (6.4%)	27 (15.6%)	56 (9.0%)	
Clinical, A ≥ 2, B ≥ 4, C ≥ 1	18 (4.0%)	33 (19.1%)	51 (8.2%)	
Missing, *n*	18	7	25	

*Note*. Binary variables are presented as valid percentages (V%). Descriptives for normally distributed numeric variables are presented as means (*M*) with standard deviations (*SD*) and non-normally distributed numeric variables are presented as medians (*Mdn*) and quartiles (Q1-Q3). ADHD = attention deficit hyperactivity disorder, LD = learning disorder, Dx = diagnosis, Psychiatric Dx = previous depression, panic, social phobia, burnout, and obsessive-compulsive disorder diagnoses; GD = gambling disorder, SUD = substance use disorder, AUDIT-C = Alcohol Use Disorder Identification Test-Consumption, BSMAS = Bergen Social Media Addiction Scale, PHQ-9 = Patient Health Questionnaire-9, GAD-7 = Generalize Anxiety Disorder-7 scale, s-ES = Gothenburg Institute of Stress Medicine’s Self-Reported Exhaustion Syndrome. *At-risk burnout positive if participants score 1/2 in part A and 3/6 in part B, or, 2/2 in part A and 3/6 in part B, or, 1/2 in part A and 4+/6 in part B

### Mental health outcomes

**Fig. 1:**
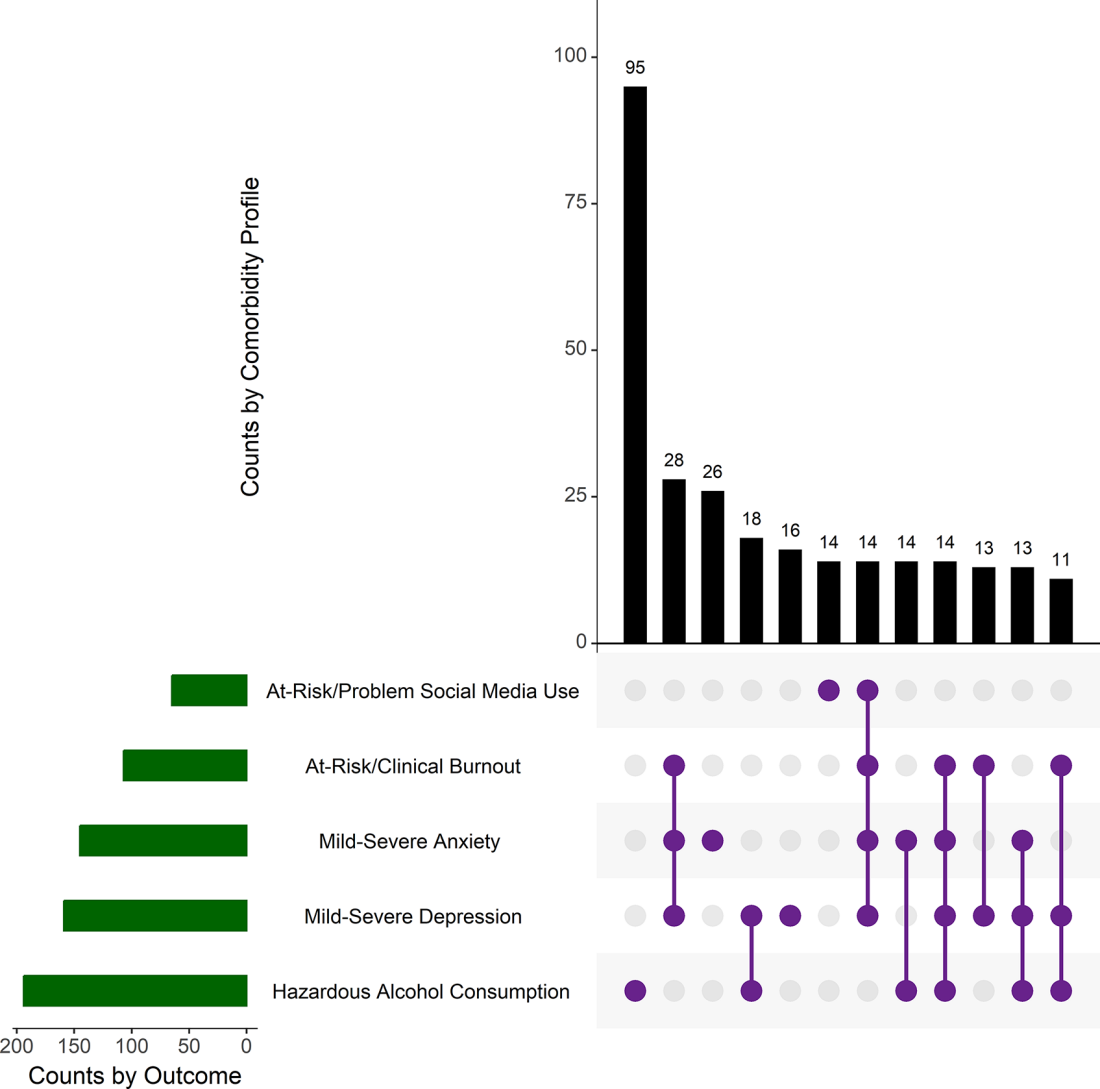
UpSet plot of comorbidity profiles

Including alcohol screenings, 41.1% of participants screened positive for a moderate-to-severe mental health condition versus 14.7% when excluding alcohol screenings. The most common comorbidity profiles when assessing all five outcomes simultaneously after pairwise deletion were no elevated symptoms (59.0%), hazardous alcohol consumption (26.4%), burnout (2.9%), hazardous alcohol consumption and burnout (1.4%), moderate-to-severe depression and anxiety as well as burnout (1.2%), followed by moderate-to-severe depression and burnout (0.9%). The remaining 10 combinations accounted for 8.2% of all cases. Cases of mental health outcomes with symptom burden ranging from mild and at-risk symptom burden to moderate and severe are presented in Fig. 1.

#### Note

*N* = 622. Only the 12 most common comorbidity profiles are presented to retain anonymity of participants. Dots represent whether outcomes were reported individually (single dot) or in conjunction with others (connected dots). Vertical bars indicate the number of cases corresponding to each combination, while horizontal bars illustrate the cumulative frequency for each of the five outcomes. At-Risk/Problem Social Media Use = BSMAS ≥ 14. At-Risk/Clinical Burnout = Part A 1 or 2 & Part B 3 + of s-ES. Mild-Severe Anxiety = GAD-7 ≥ 5. Mild-Severe Depression = PHQ-9 ≥ 5. Hazardous Alcohol Consumption = AUDIT-C ≥ 6 (Males) or 4 (Females).

### Cross-study comparisons

We adjusted respective scale cut-offs for mental health symptoms to enable cross-study comparisons. The point prevalence of elevated mental health symptoms differed in our sample compared to other athlete and general population samples. A higher percentage of our sample screened positively for hazardous drinking than a sample of Swedish national team athletes [10], χ^2^(1)_*male*_ = 16.74, *p* < .001, χ^2^(1)_*female*_ = 6.21, *p* = .01. Comparatively, only our male sample endorsed more hazardous drinking than a large sample of occupational or health-related employees in Sweden [60], χ^2^(1) = 26.98, *p* < .001. Likewise, only our male sample was less likely to screen positively for problematic social media use than a sample of 990 male participants ages 16 and older from the general population in Sweden [42], *p* = .001. A smaller proportion of our male sample screened positively for moderate-to-severe depression, *p* = .004, and moderate-to-severe anxiety, *p* = .004, than the Swedish national team-sport male athlete sample [10] but also less likely than a community male sample from Stockholm [50], χ^2^(1)_*depression*_ = 22.41, *p* < .001, χ^2^(1)_*anxiety*_ = 21.11, *p* < .001. Lastly, a larger proportion of our female sample screened positively for burnout than a community sample from southern Sweden [53], χ^2^(1) = 11.70, *p* < .001. Differences across studies are illustrated in Table 2.

**Table 2:** Cross-study comparisons of mental health outcome prevalence rates

Outcome	Measure & Cutoff	Present Study	Comparison Studies	
**Hazardous Alcohol Consumption**			Swedish National Team Athletes^*a*^	Employed Adults in Sweden^*b*^
Male	AUDIT-C ≥ 5	47.5%	27.7%**	35.7%**
Female	AUDIT-C ≥ 4	36.4%	24.5%*	30.7%
**Problematic Social Media Use**			General Population Sample in Sweden^*c*^	USA, UK, NZ, AUS Adult Sample^*d*^
Male	BSMAS ≥ 19	1.1%	4.3%**	
	BSMAS ≥ 26	0.0%		1.0%^*NA*^
Female	BSMAS ≥ 19	8.7%	5.7%	
	BSMAS ≥ 26	1.2%		1.1%
**Moderate-Severe Depression**			Swedish National Team Athletes^*a*^	Residents of Sweden^*e*^
Male	PHQ-9 ≥ 10	1.6%	6.6%**	8.2%**
Female	PHQ-9 ≥ 10	15.0%	20.4%	12.9%
**Moderate-Severe Anxiety**			Swedish National Team Athletes^*a*^	Residents of Sweden^*e*^
Male	GAD-7 ≥ 10	1.6%	6.5%**	
	GAD-7 ≥ 8	3.1%		10.6%**
Female	GAD-7 ≥ 10	10.4%	16.8%	
	GAD-7 ≥ 8	20.8%		17.9%
**Clinical Burnout**			Employed Adults in Sweden^*f*^	
Male	s-ES	4.0%	4.9%	
Female	s-ES	19.1%	9.9%**	

Note^*a*^ Åkesdotter et al. (2020), ^*b*^ Hallgren et al. (2021), ^*c*^ Henzel & Håkansson (2021), ^*d*^ Zarate et al. (2023), ^*e*^ Johansson et al. (2013), ^*f*^ Persson et al. (2016). AUDIT-C = Alcohol Use Disorder Identification Test-Consumption, BSMAS = Bergen Social Media Addiction Scale, PHQ-9 = Patient Health Questionnaire-9, GAD-7 = Generalize Anxiety Disorder-7 scale, s-ES = Gothenburg Institute of Stress Medicine’s Self-Reported Exhaustion Syndrome. NA = not applicable due to zero cases in the present study. USA, UK, NZ, AUS = United States of America, United Kingdom, New Zealand, Australia. *p < .05, **p < .01

### Effect of concussive events on symptom burden

**Fig. 2:**
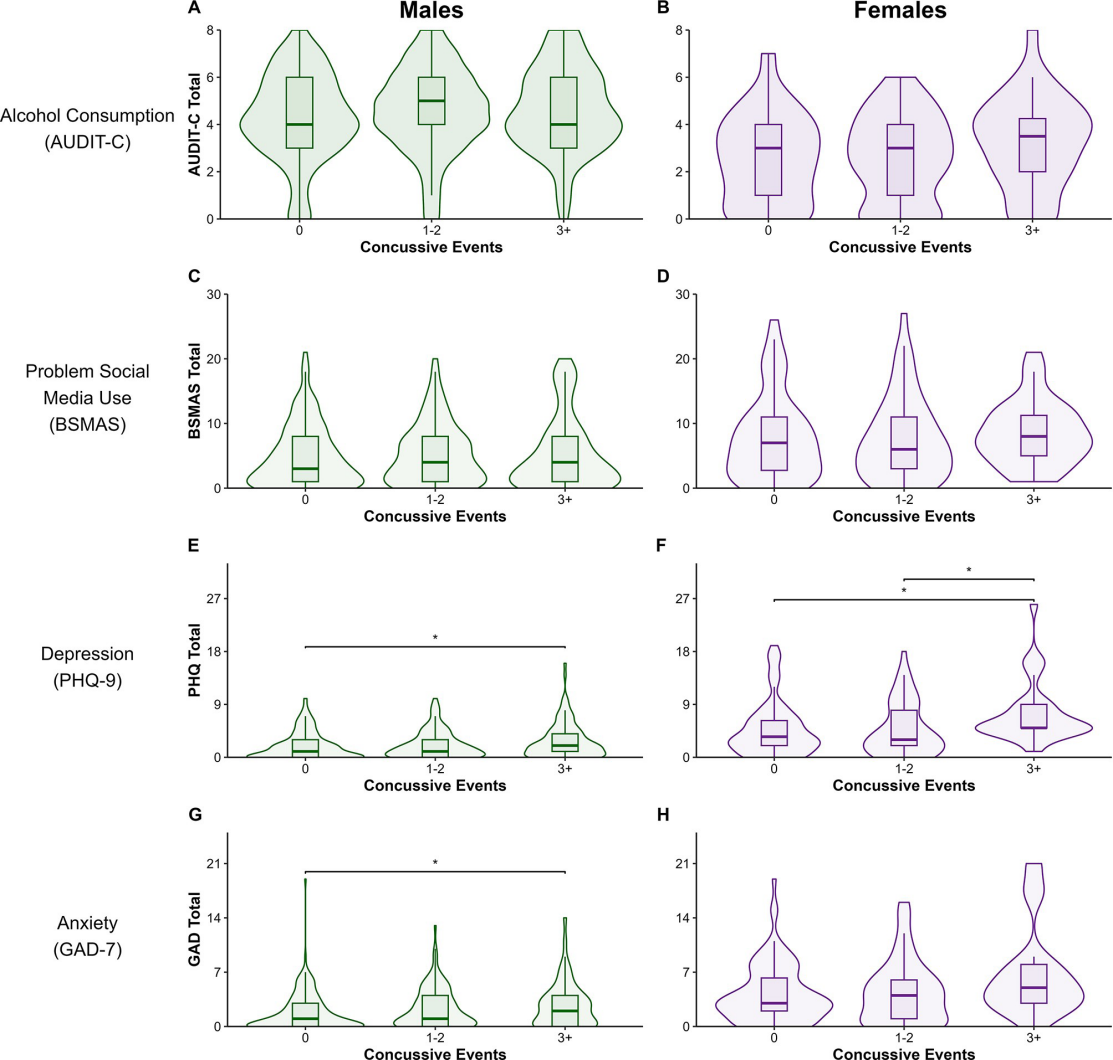
Effect of concussive events on mental health symptom burden by sex

There were significant differences in PHQ-9 scores for both males, *KW*χ(2) = 9.04, *p* = .01, and females, *KW*χ(2) = 12.06, *p* = .002. Male athletes with 3 + concussive events had greater PHQ scores (*Mdn* = 2 [1–4]) than those with none (*Mdn* = 1 [0–3]), *p*_*adj*_ = 0.008, while female athletes with 3 + impacts had higher scores (*Mdn* = 5 [5–9]) than those with none (*Mdn* = 4 [2–6]), *p*_*adj*_ = 0.004, and those with 1–2 (*Mdn* = 3 [2–8]), *p*_*adj*_ = 0.006. Lastly, among males were there differences in GAD scores, *KW*χ(2) = 12.26, *p* = .002. Male athletes with 3 + impacts had higher scores (*Mdn* = 2 [0–4]) than those with no impacts (*Mdn* = 1 [0–3]), *p*_*adj*_ = 0.003. Female participants with 3 + concussive events trended toward endorsing greater GAD scores (*Mdn* = 5 [3–8]) than those with no impacts (*Mdn* = 3 [2–6]), *p*_*adj*_ = 0.06. Relationships across all continuous outcomes are illustrated in Fig. 2. A sensitivity analysis was conducted by splitting the concussive impact variable into four groups (0, 1–2, 3–5, 6+). Results showed that only males with 6 + concussive events had higher PHQ and GAD scores compared to those with none, and higher BSMAS scores compared to those with 3–5. Detailed results are provided in Additional file 2 (Figure A1).

#### Note

Overlayed box and violin plots. (A) AUDIT-C scores compared using ANOVA for males and (B) females. (C) Kruskal-Wallis and Dunn tests comparing BSMAS scores for males and (D) females. (C) Kruskal-Wallis and Dunn tests comparing PHQ-9 scores for males and (F) females. (G) Kruskal-Wallis and Dunn tests comparing GAD-7 scores for males and (H) females. AUDIT-C = Alcohol Use Disorder Identification Test – Consumption. BSMAS = Bergen Social Media Addiction Scale, PHQ-9 = Patient Health Questionnaire-9, GAD-7 = Generalize Anxiety Disorder-7 scale. *p < .05.

We deployed logistic regressions to assess the relationship between concussive events and screening positive for being at-risk or presenting with mild-to-severe levels of mental health outcomes adjusted for ADHD/learning disorder diagnoses, age, and sex. When adjusting for covariates, athletes who reported 3 + concussive events had 2.1 [95% CI 1.2–3.8] times higher odds of endorsing elevated (at least mild) depressive symptoms than those with none, *p*_*adj*_ = 0.005, and 2.1 [95% CI 1.1–3.9] times higher odds than those who reported 1–2 concussive events, *p*_*adj*_ = 0.02. In addition, those reporting 3 + concussive events had 3.5 [95% CI 1.8–6.9] times higher odds of endorsing at-risk to clinical burnout than those with none, *p*_*adj*_ < 0.001, and 2.0-fold [95% CI 1.0–4.0] higher odds than those with 1–2 concussive events, *p*_*adj*_ = 0.04. Controlling for covariates and treating CHx as a continuous predictor, CHx was positively associated with at-risk/problematic social media use (*p* = .03), mild-to-severe depression (*p* < .001), mild-to-severe anxiety (*p* = .01), and at-risk and clinical burnout (*p* < .001). The CHx and sex interaction term was only significant in models of problematic social media use, where the effect of CHx was significantly attenuated among females (*p* = .003). Table 3 summarizes the unadjusted and adjusted ORs for the variables predicting elevated mental health symptom burden.

**Table 3:** Binary logistic models predicting screening for elevated mental health symptoms

CHx	Crude OR [95% CI]	Adjusted OR [95% CI]
	Hazardous Alcohol Consumption	
0 vs. 1–2	1.15 [0.71–1.86]	1.17 [0.72–1.91]
1–2 vs. 3+	1.22 [0.71–2.11]	1.30 [0.75–2.25]
0 vs. 3+	1.40 [0.86–2.30]	1.52 [0.92–2.52]
Continuous	1.05 [0.96–1.15]	1.07 [0.98–1.17]
CHx*Female	1.08 [0.88–1.32]	1.07 [0.87–1.31]
	At-Risk/Problematic Social Media Use (BSMAS ≥ 14)	
0 vs. 1–2	1.19 [0.57–2.50]	1.19 [0.56–2.52]
1–2 vs. 3+	1.17 [0.52–2.63]	1.25 [0.55–2.86]
0 vs. 3+	1.40 [0.66–2.94]	1.49 [0.70–3.21]
Continuous	**1.13 [1.00-1.26]**	**1.14 [1.01–1.29]**
CHx*Female	**0.64 [0.45–0.86]**	**0.62 [0.44–0.83]**
	Mild-Severe Depression (PHQ ≥ 5)	
0 vs. 1–2	1.05 [0.62–1.78]	1.04 [0.59–1.82]
1–2 vs. 3+	1.70 [0.95–3.02]	**2.07 [1.10–3.88]**
0 vs. 3+	**1.78 [1.06–2.98]**	**2.14 [1.21–3.78]**
Continuous	**1.15 [1.05–1.27]**	**1.20 [1.08–1.32]**
CHx*Female	1.22 [0.98–1.54]	1.18 [0.94–1.50]
	Mild-Severe Anxiety (PHQ ≥ 5)	
0 vs. 1–2	1.59 [0.94–2.70]	1.60 [0.92–2.78]
1–2 vs. 3+	1.00 [0.56–1.79]	1.09 [0.59–2.01]
0 vs. 3+	1.59 [0.92–2.76]	1.74 [0.97–3.12]
Continuous	**1.13 [1.03–1.24]**	**1.14 [1.03–1.26]**
CHx*Female	1.06 [0.86–1.32]	1.04 [0.84–1.29]
	At-Risk/Clinical Burnout	
0 vs. 1–2	1.70 [0.91–3.18]	1.73 [0.89–3.37]
1–2 vs. 3+	1.65 [0.88–3.08]	**2.03 [1.02–4.04]**
0 vs. 3+	**2.80 [1.53–5.12]**	**3.52 [1.80–6.86]**
Continuous	**1.25 [1.13–1.38]**	**1.29 [1.16–1.45]**
CHx*Female	1.04 [0.83–1.31]	1.01 [0.80–1.28]

*Note*. Adjusted odds ratios controlled for age, sex, and ADHD/LD diagnoses. Listwise deletion deployed for missing data. CHx = history of concussive events, AUDIT-C = Alcohol Use Disorder Identification Test-Consumption, BSMAS = Bergen Social Media Addiction Scale, PHQ-9 = Patient Health Questionnaire, GAD-7 = Generalized Anxiety Disorder Questionnaire, s-ES = Self-Reported Exhaustion Syndrome Scale. Bold face indicates statistical significance after Tukey corrections for multiple comparisons, *p* < .05

## Discussion

In a sample of elite ice hockey players, we compared the point prevalence rates of various mental health outcomes with other athlete and general population samples and estimated the association between self-reported concussive events and symptom burden. Hazardous alcohol consumption, often endorsed in tandem with depression and anxiety, markedly exceeded rates observed in other elite athlete samples [10], and even the general population for male athletes [60]. In contrast, our male sample was less likely to endorse problematic social media use than male general population samples [41, 42] and less likely to self-report moderate-to-severe symptoms of depression and anxiety when compared to other male elite athlete [10] and Swedish male general population samples [50]. Meanwhile, our female sample reported higher prevalence of clinical burnout symptoms than a general population sample [53]. These findings align with studies reporting heavy alcohol use among elite athletes but contrast other studies suggesting mental health symptoms are equally prevalent in elite athletes and the general population [3, 10–12].

Male athletes in our sample reported fewer mental health symptoms overall compared to other athlete and general population samples, except for hazardous alcohol consumption, whereas most mental health outcomes were similarly or more prevalent in our female sample. Socioeconomic status and workload differences, stemming from salary discrepancies and the necessity for secondary employment between male and female leagues, may explain these differences, especially in clinical burnout symptoms [61]. Additionally, male athletes might be less likely to report adverse mental health symptoms, not necessarily due to their absence, but due to the stigma associated with expressing mental health concerns in a hypermasculine environment [5]. Similarly, pride in belonging to a microculture that glorifies alcohol overconsumption in ice hockey may explain the heightened prevalence of hazardous drinking among both male and female athletes [6].

The notably high prevalence of hazardous drinking within this cohort is concerning, as it poses immediate risks to these athletes and may exacerbate long-term health consequences [33–36]. Excessive alcohol consumption is a significant risk factor for mortality and is associated with the development of numerous chronic health conditions, including neuropsychiatric disorders, later in life [62, 63]. Some research on former elite athletes may present a more nuanced picture. Notably, Kontro and colleagues [64, 65] found little evidence that former male elite athletes in Finland were at heightened risk of alcohol-related hospitalizations compared to matched controls or mortality compared to their own brothers. In a similar study by the same group, it was demonstrated that although former elite athletes reported significantly higher alcohol use in 1985 compared to age- and region-matched controls, their consumption was not significantly higher 10, 16, or 23 years later [66]. Moreover, a recent systematic review conducted by Munro and colleagues [67], examining the relationship between alcohol use and both cognitive and neuropathological outcomes in athletes with a history of mild traumatic brain injury, concluded that there is limited evidence supporting such an association. Although excessive alcohol use is detrimental to health, it is possible that elite athlete populations may reduce their drinking later in life and retain certain healthy behaviors that could protect them from developing alcohol-related chronic conditions [65, 66].

Similar to previous studies utilizing large samples of retired and active athletes, sustaining three or more concussive events was associated with elevated (at-least mild) symptoms of depression [18, 20–23]. Similarly, those who reported three or more concussive events were more likely to be at-risk or have clinical burnout symptoms, which has not been previously described in the literature to the authors’ knowledge. Modeling concussive events as a continuous predictor revealed positive correlations with all outcomes except for hazardous alcohol consumption, the latter of which is in line with a recent study in former professional American football players [68]. Interaction models indicated that sex differences were minimal, though the effect of concussive events on at-risk/problematic social media use was only observed in males. This was unexpected as previous research indicates that females tend to experience more severe mood-related post-concussive sequelae compared to males [24, 25], though may be the result of our nuanced approach of using subclinical thresholds for these analyses. Importantly, the vast majority of cases were in the mild range for each outcome (e.g., PHQ-9 scores between 5 and 9).

As the present sample consisted of only active athletes, the presence of these symptoms may be the result of evolving neurophysiological processes induced by recurrent concussive injuries [15, 16]. Axonal injury and the impaired integrity of white matter tracts may be one such injury mechanism that impairs neuropsychological function as highlighted by a recent neuroimaging study employing advanced diffusion imaging techniques in active athletes with post-concussive symptoms [69]. A plethora of demographic, psychosocial, and behavioral factors may also contribute to the manifestation and persistence of these symptoms over time. Pre-existing mental health issues, isolation, deconditioning, poor sleep, inadequate injury management, negative psychological responses, and pressures from influential figures and cultural norms valuing “toughness” can all contribute to the worsening of mental health in concussed athletes and interfere with effective concussion treatment [9, 21, 70].

Considering this, our findings reinforce the association between recurrent concussive injuries and elevated (at least mild) depressive symptoms, and also introduce burnout symptoms as potential sequelae. While the correlations between CHx as a continuous variable with both at-risk/problematic social media use and mild-to-severe anxiety were statistically significant, these findings do not necessarily imply clinical relevance but do warrant further investigation. Furthermore, we cannot determine the temporal progression of these symptoms—whether they were pre-existing, exacerbated by injury, persisted after the most recent injury, or reemerged following initial recovery from previous concussions. Nevertheless, routine mental health screening and monitoring beyond the acute phase of injury may uncover untreated psychiatric symptoms and prove beneficial in providing optimal care to elite athletes.

### Strengths and limitations

This study contributes to the literature by examining the effects of concussive events on the mental health symptom burden of current elite ice hockey players using a larger sample size than previous studies in the field. In contrast to other studies, we were able to control for pertinent covariates when assessing relationships. In addition, our sample comprised all but one of the teams competing in Sweden’s top ice hockey leagues for both male and female athletes. This enhanced the internal and external validity of our study and improved our understanding of the relationships throughout Sweden. Furthermore, to the best of the authors’ knowledge, our study was the first to examine the association between CHx and other unexplored mental health outcomes, such as burnout and social media use. Our findings provide a benchmark against which future research can be built and compared.

Importantly, the present study’s findings should be interpreted commensurate with its methodological and statistical limitations. Imprimis, the cross-sectional nature of this study does not allow us to assess causality. Despite this, our study serves as a steppingstone from which future studies employing prospective study designs can base their investigation on. Secondly, due to our sample size, we were unable to control for more covariates than what was statistically viable. This is still an improvement over other studies, which were unable or decided not to include covariates in their models. Thirdly, the data collection process spanned seven months for the male sample, which may have introduced seasonal variation into the data we collected. For instance, symptom burden may vary between participants recruited during pre-season, at the beginning of the season, and during the winter holidays [22]. Distributing the survey via online channels over a shorter span rather than in-person over several months may have minimized the amount of seasonal variation, but likely would have resulted in poorer response rate. Fourth, the cut-offs used in our survey to indicate subclinical and clinical levels of symptom burden may impact our interpretations and comparisons with other populations. For example, a cut-off score of 10 on the PHQ-9 has been shown to overestimate the prevalence of moderate-to-severe depressive symptoms in the general population [71]. Similarly, the “at-risk” threshold for burnout has not been validated to identify individuals who are at risk. However, as highlighted by Persson et al. [53], the s-ES identifies a smaller proportion of participants exhibiting burnout symptoms compared with other measures, so opting for a more liberal cut-off as we did may compensate for the inherent conservativeness of the s-ES. Likewise, our measurements have not been validated specifically for Swedish athletic populations, which may introduce information bias. Again, this was a trade-off, as there are few existing mental health symptom batteries that have both been implemented and validated in Swedish athletes. Therefore, we preferred measures that had already been used in previous research based in Sweden.

Although our sample consisted of participants from 37 of 38 teams, over one-third of all players who competed during the season either did not participate or declined, introducing a degree of selection bias. Due to mental health issues, those with poorer mental health may have been more likely to decline participation or be absent from team meetings. Most importantly, we did not collect data pertaining to how long ago participants sustained their most recent concussive event, which made it possible, albeit unlikely, for a participant to complete the survey during the acute phase of injury, thereby confounding our goal of analyzing post-acute effects of concussive events. Lastly, our findings may not generalize to athletes in different sports or those competing at lower levels given the inherent heterogeneity in athletes’ sports and competition levels.

## Conclusions

The prevalence of elevated adverse mental health symptoms among elite ice hockey players differed from that observed in other athlete and general population samples, highlighting potential avenues for intervention and prevention. Our results corroborate previous studies showing a relationship between concussive events and augmented (at least mild) symptoms of depression and introduce a connection with burnout, though prospective studies are needed to assess the causal link. Regular mental health screening and monitoring beyond the acute injury phase may support clinicians in providing adequate treatment for concussed elite athletes.

## Electronic supplementary material

Below is the link to the electronic supplementary material.

Supplementary Material 1

Supplementary Material 2

## Data Availability

In accordance with our ethical permit, data are not able to be shared due to the recognizability of participating athletes.

## Abbreviations

ADHD: attention deficit hyperactivity disorder
ANOVA: analysis of variance
AUDIT-C: Alcohol Use Disorder Identification Test – Consumption
BSMAS: Bergen Social Media Addiction Scale
CHx: concussion history
CI: confidence interval
GAD-7: Generalized Anxiety Disorder questionnaire – 7
HA: HockeyAllsvenskan
KW: Kruskal-Wallis test
LD: learning disorder
M: mean
Mdn: median
OR: odds ratio
PHQ-9: Patient Health Questionnaire – 9
Q1-Q3: quartile one to quartile three
s-ES: Stress Medicine’s Self-Reported Exhaustion Syndrome
SICO: Swedish Ice-Hockey Players’ Union [Sveriges Ishockeyspelares Centralorganisation]
SHL: Swedish Hockey League
SWHL: Swedish Women’s Hockey League
V%: valid percent
