# Correction: Mental health symptom burden in elite ice hockey players and its association with self-reported concussive events

**DOI:** 10.1186/s13102-024-01039-5

**Published:** 2024-12-16

**Authors:** Mitchell J. Andersson, Göran Kenttä, Emma Claesdotter-Knutsson, Anders Håkansson

**Affiliations:** 1 Department of Clinical Sciences Lund, Psychiatry, Lund University, Lund, Sweden; 2 Clinical Sports and Mental Health Unit, Malmö Addiction Center, Region Skåne, Malmö, Sweden; 3 The Swedish School of Sport and Health Sciences, Stockholm, Sweden; 4 The Swedish Sports Confederation, Stockholm, Sweden; 5 School of Human Kinetics, University of Ottawa, Ottawa, ON Canada; 6 Child and Adolescent Psychiatry Outpatient Clinic, Region Skåne, Lund, Sweden

**Correction: BMC Sports Sci Med Rehabil 16**,** 197 (2024)**.

10.1186/s13102-024-00989-0.

Following publication of the original article [1], the authors identified a typesetting error, whereby Supplementary file 1 was not included. In the original article [1], supplementary file 2 was published as supplementary file 1.

The correct Supplementary file 1 and 2 are published within this correction article and the original article [1] has been corrected.

## Electronic Supplementary Material

Below is the link to the electronic supplementary material.

Supplementary Material 1

Supplementary Material 2
