# Supplementary material for: Correction: Mental health symptom burden in elite ice hockey players and its association with self-reported concussive events
Source: BMC Sports Sci Med Rehabil. 2024 Dec 16;16:241. doi: 10.1186/s13102-024-01039-5 (PMC11648289; doi:10.1186/s13102-024-01039-5)
Supplement: Supplementary file 1 — Supplementary Material 1 [file 13102_2024_1039_MOESM1_ESM.pdf]

Elitidrottande hockeyspelare, hjärnskakning och psykisk hälsa

Information to the study participants

We would like to ask you if you would like to participate in a study. In this document you will receive information about the project and what it means to partake in it.

What is this study, and why should I participate?

The purpose is to investigate how common concussions are among elite hockey players, and the connection between mental health and concussions. The study has been requested by the union (SICO), which is why you are asked to participate.
The study is conducted by Lunds University, which is the organization responsible for this research.

How is the study conducted?

As a participant you are asked to fill out a questionnaire on the pages following this introduction. The questionnaire is anonymous, no IP's are logged and there will be no way to trace who you are or what team you are a part of.
This means we will not be able to follow up on your results, but in the end of the questionnaire you will be presented with your results of all the screening tools and if they indicate risk of any mental health. You will also be given advice how to seek medical assistance if you are in distress.
The questionnaire takes about 15-20 minutes to complete, we do however urge you to take your time and answer truthfully since the result could work as an incentive to help those players who are suffering from mental disorders.

Study risks and privacy

Since all information will be stored anonymously and will not be able to be backtracked to a specific person, there should be no risks of participating in the study.
If you feel the need for support regarding mental health there is a unit called "Elitidrott och hälsa" (Elite athletes and health) that you can contact through 1177.se or by phone number 08-12345780.

What happens with my information?

The study will collect information about you.
The answers you give in the questionnaire will be about how many concussions you have suffered and how you see your own mental health status. All answers will be saved encrypted and will not be able to be connected to a separate individual since no personal data is requested.
Your answers and results will be treated in a way so that no unauthorized personnel will be able to take part of it. The information is only allowed to be used in the way you have accepted them to be used.

How will I be able to get the results of the study?

The results of the different screening tools will be presented for you at the end of the questionnaire. The entire study is regarding hockey-players on an elite level through the union SICO and the results will thus be presented through them but also in a medical journal. Even unforeseen finds will be presented since it could indicate that further research needs to be conducted in the field.

Insurance and compensation

Since you as a participant will not give any personal information and we do not see risks of partaking, there is no need for any insurance. If you contact Elitidrott och Hälsa you will be protected by the patient-insurance.

Participation is voluntary

Your participation is completely voluntary, and you can at any time decide to cancel your participation while filling out the questionnaire. You do not have to give a reason why you decide to not participate, and it will not affect your future possible treatment or care.

If you decide to withdraw your participation; simply close the questionnaire without entering any more data.

If you for some reason don't want to participate at this moment, but want to do it at a later time, please contact the lead investigator on the e-mail below.

Who is in charge of the study?

The study is conducted by Lunds University. The lead investigator is Björn Gunnarsson, M.D. , Helsingborg Emergency Department, who can be contacted on e-mail: InfoHockeyHjarnskakning@gmail.com, or by phone: 0706 629 081

Scientifical lead and supervisor is professor Anders Håkansson, Specialist of psychiatry, Department of addictive disorders, Malmö, who can be contacted by e-mail: anders_c.hakansson@med.lu.se

---

Consent to participate

I have received written and oral information about the study and have been given the possibility to ask questions. I get to keep a written information.

- [ ] I consent to participate in the study "Elitidrottande hockeyspelare, hjärnskakning och psykisk hälsa"

- [ ] I consent to that the information given by me is treated the way that has been described in this information.

Gender?

- ◯ Male
- ◯ Female
- ◯ Would not like to answer

Age?

- ◯ 25 years or younger
- ◯ 26 years or above

Which of the following symptoms have you experienced within a week after a blow to the head or neck?

- ☐ Have never sustained such a blow to the head/neck
- ☐ Nausea/vomiting
- ☐ Vertigo
- ☐ Foggy vision
- ☐ Difficulty of keeping balance
- ☐ Sound sensitivity
- ☐ Sensitivity to light
- ☐ Feeling of sluggish thinking
- ☐ Difficulty to concentrate
- ☐ Amnesia

- [ ] Lack of energy
- [ ] Confusion
- [ ] Drowsiness
- [ ] Been overly emotional
- [ ] Irritability
- [ ] dysphoria
- [ ] Nervousness/Anxiety
- [ ] Insomnia
- [ ] Have never experienced any of these symptoms after a blow to the head/neck

Other

How many times have you experienced head/neck trauma that resulted in:

Difficulty of maintaing balance or fogged vision WITHOUT being unconscious or having amnesia.

- ○ Never
- ○ Once
- ○ Twice
- ○ Thrice
- ○ On four or more occasions

A short period of amnesia but no lack of consciousness.

- Never
- Once
- Twice
- Thrice
- On four or more occasions

Lack of consciousness shorter than 30 seconds

- Never
- Once
- Twice
- Thrice
- On four or more occasions

Lack of consciousness for longer than 30 seconds.

- Never
- Once
- Twice
- Thrice
- On four or more occasions

How many times has a head/neck trauma resulted in:

That you have been forced out of play for the current game, but have been allowed to play the next one

- ◯ Never
- ◯ Once
- ◯ Twice
- ◯ Thrice
- ◯ On four or more occasions

That you have been taken out of play for more than one game

- ◯ Never
- ◯ Once
- ◯ Twice
- ◯ Thrice
- ◯ On four or more occasions

Match penalty/game misconduct for the opponent

- ◯ Never
- ◯ Once
- ◯ Twice
- ◯ Thrice
- ◯ On four or more occasions

Are you diagnosed (or have previously been diagnosed) with any of the following:

Learning disabilities (Dyslexia, dyscalculia)

- [ ] Yes, within a year after being concussed.
- [ ] Yes, but not within a year after being concussed.
- [ ] No

Depression

- [ ] Yes, within a year after being concussed.
- [ ] Yes, but not within a year after being concussed.
- [ ] No

Panic disorder

- [ ] Yes, within a year after being concussed.
- [ ] Yes, but not within a year after being concussed.
- [ ] No

Social phobia

- [ ] Yes, within a year after being concussed.
- [ ] Yes, but not within a year after being concussed.
- [ ] No

ADD/ADHD

- [ ] Yes, within a year after being concussed.
- [ ] Yes, but not within a year after being concussed.
- [ ] No

Chronic fatigue syndrome

- [ ] Yes, within a year after being concussed.
- [ ] Yes, but not within a year after being concussed.
- [ ] No

OCD

- [ ] Yes, within a year after being concussed.
- [ ] Yes, but not within a year after being concussed.
- [ ] No

Gambling addiction

- [ ] Yes, within a year after being concussed.
- [ ] Yes, but not within a year after being concussed.
- [ ] No

Substance addiction

- [ ] Yes, within a year after being concussed.
- [ ] Yes, but not within a year after being concussed.
- [ ] No

The following questions are regarding gambling for money:

Considering the past 12 months, have you bet more than you could really afford to lose?

- ◯ Never
- ◯ Sometimes
- ◯ Most of the time
- ◯ Always

Considering the past 12 months, have you needed to gamble with larger amounts of money to get the same feeling of excitement?

- ◯ Never
- ◯ Sometimes
- ◯ Most of the time
- ◯ Always

Considering the past 12 months, have you gone back on another day to try to win back the money you lost?

- ◯ Never
- ◯ Sometimes
- ◯ Most of the time
- ◯ Always

Considering the past 12 months, have you borrowed money or sold anything to gamble?

- ◯ Never
- ◯ Sometimes
- ◯ Most of the time
- ◯ Always

Considering the past 12 months, have you felt that you might have a problem with gambling?

- Never
- Sometimes
- Most of the time
- Always

Considering the past 12 months, have people criticised your betting or told you that you had a gambling problem, whether or not you thought it was true?

- Never
- Sometimes
- Most of the time
- Always

Considering the past 12 months, have you felt guilty about the way you gamble or what happens when you gamble?

- Never
- Sometimes
- Most of the time
- Always

Considering the past 12 months, has gambling caused you any health problems, including stress or anxiety?

- Never
- Sometimes
- Most of the time
- Always

Considering the past 12 months, has your gambling caused any financial problems for you or your household?

- ◯ Never
- ◯ Sometimes
- ◯ Most of the time
- ◯ Always

How often did you have a drink containing alcohol in the past year?

- ◯ Never
- ◯ Monthly or less
- ◯ 2-4 times a month
- ◯ 2-3 times per week
- ◯ 4 or more times a week

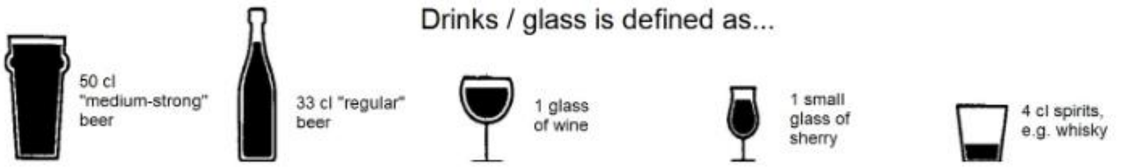

How many drinks containing alcohol did you have on a typical day when you were drinking in the past year?

- ◯ 1-2
- ◯ 3-4
- ◯ 5-6
- ◯ 7-9
- ◯ 10 or more

How often did you have 4 (female)/5 (male) or more drinks on one occasion in the past year?

- ◯ Never
- ◯ Monthly or less
- ◯ Every month
- ◯ Every week
- ◯ Daily or almost daily

Have you at any time during the last year taken any of the following substances:

Cannabis

- ◯ Yes
- ◯ No
- ◯ I'd rather not answer

Benzodiazepines

- ◯ Yes
- ◯ No
- ◯ I'd rather not answer

Cocaine

- ◯ Yes
- ◯ No
- ◯ I'd rather not answer

Amphetamine/MDMA or ADHD-medication without having a prescription (Ritaline, Elvanse etc)

- ◯ Yes
- ◯ No
- ◯ I'd rather not answer

Painkillers without having a prescription (Morphine, Codeine, OxyContine etc)

- ◯ Yes
- ◯ No
- ◯ I'd rather not answer

Anabolic steroids

- ◯ Yes
- ◯ No
- ◯ I'd rather not answer

Have you used any medication for troubled sleeping lately:

- ◯ Yes, I use it almost daily
- ◯ Yes, the last week
- ◯ Yes, the last month
- ◯ No
- ◯ I'd rather not answer

If yes: which substance?

[ ]

Over the last 2 weeks, how often have you been bothered by the following problems?

Feeling nervous, anxious, or on edge

- ◯ Not at all
- ◯ Several day
- ◯ More than half the days
- ◯ Nearly every day

Not being able to stop or control worrying

- ◯ Not at all
- ◯ Several day
- ◯ More than half the days
- ◯ Nearly every day

Worrying too much about different things

- ◯ Not at all
- ◯ Several day
- ◯ More than half the days
- ◯ Nearly every day

Trouble relaxing

- ◯ Not at all
- ◯ Several day
- ◯ More than half the days
- ◯ Nearly every day

Being so restless that it's hard to sit still

- ◯ Not at all
- ◯ Several day
- ◯ More than half the days
- ◯ Nearly every day

Becoming easily annoyed or irritable

- ◯ Not at all
- ◯ Several day
- ◯ More than half the days
- ◯ Nearly every day

Feeling afraid as if something awful might happen

- ◯ Not at all
- ◯ Several day
- ◯ More than half the days
- ◯ Nearly every day

How difficult have these problems made it to play ice-hockey, do work, take care of things at home, or get along with other people?

- ◯ Not at all
- ◯ Somewhat difficult
- ◯ Very difficult
- ◯ Extremely difficult

Over the last 2 weeks, how often have you been bothered by the following problems?

Little interest or pleasure in doing things?

- ◯ Not at all
- ◯ Several days
- ◯ More than half the days
- ◯ Nearly every day

Feeling down, depressed, or hopeless?

- ◯ Not at all
- ◯ Several days
- ◯ More than half the days
- ◯ Nearly every day

Trouble falling or staying asleep, or sleeping too much?

- ◯ Not at all
- ◯ Several days
- ◯ More than half the days
- ◯ Nearly every day

Feeling tired or having little energy?

- ◯ Not at all
- ◯ Several days
- ◯ More than half the days
- ◯ Nearly every day

Poor appetite or overeating?

- ◯ Not at all
- ◯ Several days
- ◯ More than half the days
- ◯ Nearly every day

Feeling bad about yourself — or that you are a failure or have let yourself or your family down?

- ◯ Not at all
- ◯ Several days
- ◯ More than half the days
- ◯ Nearly every day

Trouble concentrating on things, such as reading the newspaper or watching television?

- ◯ Not at all
- ◯ Several days
- ◯ More than half the days
- ◯ Nearly every day

Moving or speaking so slowly that other people could have noticed? Or so fidgety or restless that you have been moving a lot more than usual?

- ◯ Not at all
- ◯ Several days
- ◯ More than half the days
- ◯ Nearly every day

Thoughts that you would be better off dead, or thoughts of hurting yourself in some way?

- ◯ Not at all
- ◯ Several days
- ◯ More than half the days
- ◯ Nearly every day

How difficult have these problems made it to play ice-hockey, do work, take care of things at home, or get along with other people?

- ◯ Not at all
- ◯ Somewhat difficult
- ◯ Very difficult
- ◯ Extremely difficult

1. Have you felt mentally and/or physically exhausted for more than 2 weeks?

- ◯ No
- ◯ Yes

---

2. Is this fatigue coming from being under pressure for period of time that is longer than 6 months?

- ◯ No
- ◯ Yes

---

3. During the last 2 weeks, have you experienced:

Difficulties concentrating or loss of memory?

- ◯ Yes
- ◯ No

Highly reduced capability to perform whenever there is short amounts of time.

- ◯ Yes
- ◯ No

Being emotionally unstable or irritable?

- ◯ Yes
- ◯ No

Trouble sleeping?

- ◯ Yes
- ◯ No

That you've felt physically weak or that you easily become exhausted?

- ◯ Yes
- ◯ No

Physical problems such as chest pains, heart palpitations, stomach aches, vertigo, increased sensibility to sound or pain somewhere?

- ◯ Yes
- ◯ No

Have these things (question 1-3) reduced your wellbeing and level of function (performance in ice-hockey, ability to work, family life, hobbies or in other important situations)?

- ◯ Yes, a lot
- ◯ Yes, somewhat
- ◯ No, not at all

The following questions are about your habits using social media (Like Facebook, instagram or snapchat).
Considering the past 12 months, how often did you ...

... spend a lot of time thinking about or planning using social media?

- ◯ Never
- ◯ Very rarely
- ◯ Rarely
- ◯ Sometimes
- ◯ Often
- ◯ Very often

... feel an urge to use social media more and more?

- ◯ Never
- ◯ Very rarely
- ◯ Rarely
- ◯ Sometimes
- ◯ Often
- ◯ Very often

... use social media to forget about personal problems?

- ◯ Never
- ◯ Very rarely
- ◯ Rarely
- ◯ Sometimes
- ◯ Often
- ◯ Very often

... try to cut down on the use of social media without success?

- ◯ Never
- ◯ Very rarely
- ◯ Rarely
- ◯ Sometimes
- ◯ Often
- ◯ Very often

... get troubled by being prohibited from social media use?

- ◯ Never
- ◯ Very rarely
- ◯ Rarely
- ◯ Sometimes
- ◯ Often
- ◯ Very often

... negatively impacted your life as a hockey player/other parts of your life?

- ◯ Never
- ◯ Very rarely
- ◯ Rarely
- ◯ Sometimes
- ◯ Often
- ◯ Very often

Loneliness

During the last week, have you felt lonely?

- ◯ Rarely, or never (less than one day)
- ◯ Sometimes (1-2 days)
- ◯ Often (3-4 days)
- ◯ Mostly or always (5-7 days)

Own performance

How satisfied are you regarding your own performance the last 4 weeks?

- ◯ Not at all satisfied
- ◯ Somewhat satisfied
- ◯ Satisfied
- ◯ Very satisfied

Injuries

How many serious injuries have you suffered the last year? (At least 4 weeks of pause from competing or practice)

- ◯ 0
- ◯ 1-2
- ◯ 3-4
- ◯ 5 or more

Current mental health
Do you feel mentally distressed now (daily for at least 2 weeks), that you have difficulties functioning normally in life/the sport?

- ◯ Yes
- ◯ No

Previous mental health
Have you ever felt so mentally distressed (daily for at least 2 weeks) that you have felt difficulties funtioncing normally in life/the sport?

- ◯ Yes
- ◯ No

Elitidrottande hockeyspelare, hjärnskakning och psykisk hälsa

The results above are based only on screening tools and can only indicate disorders, not diagnoses. If you feel distressed you should not hesitate to consult a medical professional. Since the questionnaire is anonymous, you are recommended to contact the healthcare for further investigation and treatment if necessary.

As a member of riksidrottsförbundet on an elite level, there is a chance to get in contact with a unit specialising in mental health issues among elite athletes.
If you feel mentally distressed, you can recieve help by either contacting "mottagningen för Elitidrott och Hälsa" (the clinic for elite athletes and health), by 1177.se or by phone: 08-12345780. You can always go to your health clinic or in acute need of help – to the psychiatric emergency department.

The head researcher can be contacted at the following E-mail: InfoHockeyHjarnskakning@gmail.com
