# Supplementary material for: Correction: Mental health symptom burden in elite ice hockey players and its association with self-reported concussive events
Source: BMC Sports Sci Med Rehabil. 2024 Dec 16;16:241. doi: 10.1186/s13102-024-01039-5 (PMC11648289; doi:10.1186/s13102-024-01039-5)
Supplement: Supplementary file 2 — Supplementary Material 2 [file 13102_2024_1039_MOESM2_ESM.docx]

**Additional File 2**

**Sensitivity Analysis**

The concussive impacts variable was split into groups of four based on previous literature (0, 1-2, 3-5, 6+; [37]). Due to too few cases of 6+ concussive impacts group among females (*n* = 6), results must be interpreted with caution. Male athletes who reported 6+ concussive impacts endorsed higher BSMAS scores than those with 3-5 impacts, *p* = .047. They also reported higher PHQ-9 scores than those none, *p* = .02., while for females, those reporting 3-5 impacts had higher scores than those with none, *p* = .02 and 1-2 impacts, *p* = .02. Lastly, male athletes endorsed higher GAD-7 scores than those with none, *p* = .005. All results are illustrated in Figure 1S.

**Figure 1S**

*Effect of concussive impacts on mental health symptom burden by sex (Sensitivity Analysis)*


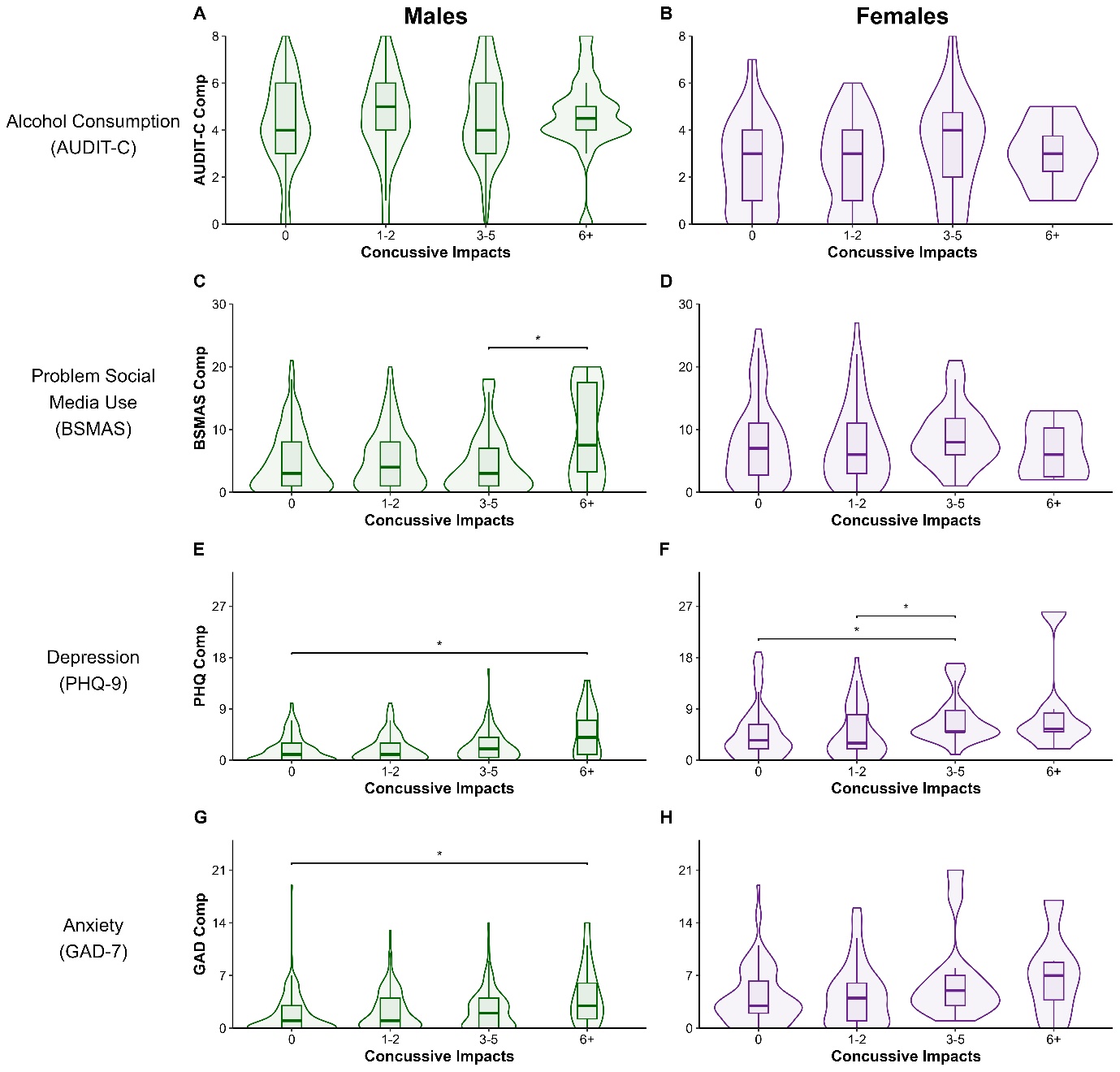


*Note*. (A) AUDIT-C scores compared using ANOVA for males and (B) female. (C) Kruskal-Wallis and Dunn tests comparing BSMAS scores for males and (D) females. (C) Kruskal-Wallis and Dunn tests comparing PHQ-9 scores for males and (F) females. (G) Kruskal-Wallis and Dunn tests comparing GAD-7 scores for males and (H) females. AUDIT-C = Alcohol Use Disorder Identification Test – Consumption. BSMAS = Bergen Social Media Addiction Scale, PHQ-9 = Patient Health Questionnaire-9, GAD-7 = Generalize Anxiety Disorder-7 scale. **p* < .05.
